# Genome Analyses of Ten New Ape Adenoviruses with Similarity to Human *Mastadenovirus* C

**DOI:** 10.3390/ijms23179832

**Published:** 2022-08-30

**Authors:** Selas T. F. Bots, Vera Kemp, Iris J. C. Dautzenberg, Rob C. Hoeben

**Affiliations:** Department of Cell and Chemical Biology, Leiden University Medical Center, 2333 ZC Leiden, The Netherlands

**Keywords:** simian adenovirus, Mastadenovirus, HAdV-C, recombination, non-human primates, taxonomy

## Abstract

The adenoviruses (AdVs) isolated from humans are taxonomically grouped in seven different species in the Mastadenovirus genus (HAdV-A through G). AdVs isolated from apes are often included in one of the human AdV species. Here we describe the sequence analyses of ten new AdVs that are related to the HAdV-C species and that were isolated from healthy western lowland gorillas, bonobos, chimpanzees, and orangutans kept in Dutch zoos. We analyzed these viruses and compared their genome sequences to those of human- and ape-derived AdV sequences in the NCBI GenBank database. Our data demonstrated that the ape-derived viruses clustering to HAdV-C are markedly distinct from the human HAdV-C species in the size and nucleotide composition (%GC) of their genome, differ in the amino-acid sequence of AdV proteins, and have longer RGD-loops in their penton-base proteins. The viruses form three well-separated clades (the human, the gorilla, and the combined group of the bonobo and chimpanzee viruses), and we propose that these should each be given species-level ranks. The Ad-lumc005 AdV isolated from orangutans was found to be very similar to the gorilla AdVs, and bootstrap inference provided evidence of recombination between the orangutan AdV and the gorilla AdVs. This suggests that this virus may not be a genuine orangutan AdV but may have been transferred from a gorilla to an orangutan host.

## 1. Introduction

Human adenoviruses (HAdVs) are common viruses that are associated with a range of clinical manifestations such as upper respiratory tract infections, and gastrointestinal, ophthalmologic, and genitourinary diseases [1,2]. In immune-competent individuals the infections are usually self-limited but in immune-compromised patients (e.g., bone-marrow transplant recipients) the infection can have lethal consequences. HAdVs are relatively common, and prevalence of neutralizing immunity exceeds 60% in each of the most common types, HAdV species C type 1 (HAdV-C1), HAdV-C2, C5, A12, A31, and B3 in the Belgian population [3]. Certain HAdVs are commonly shed by humans (e.g., HAdV-F) and therefore their presence can be used as an indicator for monitoring human feces contamination in, for instance, groundwater and recreational beaches [4].

Adenoviruses (AdVs) were first identified in humans as transmissible agents responsible for the degeneration of cell cultures established from adenoidal tissue by Rowe et al. [5]. AdVs are non-enveloped and have a linear DNA genome of 35–37 kb in length. To date, AdVs are frequently used as probes for studying cellular processes such as replication, transcription, and transformation. A factor contributing to their popularity among researchers was the observation that some serotypes (e.g., HAdV-A12) can induce cancer in newborn hamsters, whereas others (e.g., HAdV-C2 and C5) are not, or are only weakly, oncogenic [6]. While these studies have been very informative, there is no direct evidence for a role of AdVs in human cancer [7]. In addition, the efficient entry of AdV particles into cells has been exploited in the use of AdVs as gene transfer vectors for experimental and clinical gene therapy and for vaccination [1,8]. For this purpose, both replication-deficient, as well as replication-competent, AdVs are being used.

Although originally identified in humans, AdVs have now also been found to occur in a range of other organisms. A wide variety of AdVs are known to replicate in birds, reptiles, and bats, and fewer AdVs are found in amphibians, fish, and turtles [9]. All these viruses are grouped in the Adenoviridae family. Within the family, six genera are recognized. Of these, the Mastadenovirus genus contains AdVs with mammalian hosts. The AdVs isolated from humans are grouped into seven ‘species’ (formerly called ‘subgroups’) within the Mastadenovirus genus, species HAdV-A through HAdV-G. New isolates are classified into ‘serotypes’ or ‘types’ based on a combination of characters including serology, hemagglutination, and restriction analyses, and more recently also whole genome sequencing, with a focus on sequence similarity [10,11]. To date, the new unique human isolates are assigned type numbers by the Human Adenovirus Working Group (http://hadvwg.gmu.edu/ accessed on 21 August 2022) from HAdV-C1 to HAdV-D113. 

The type-diversity of AdVs can increase by homologous recombination between different types within a HAdV species, which occurs relatively frequently [12,13,14]. Evidence for interspecies recombination has also been presented, although this occurs more rarely [15]. When the recombinations exchange the genes encoding the major capsid proteins penton-base, hexon, or fiber, the resulting recombinants may be more capable of evading neutralizing immunity. This could provide a selective advantage in a host population in which neutralizing immunity exists against one of the recombination partners. In addition, recombinations of the other regions E1, E3 and E4 have been described in human-derived HAdV-C viruses, although it is less evident if and how this could lead to a selective advantage [12,13].

Non-human primates also have their AdVs. While lower primates have AdVs that are relatively dissimilar from the human-derived HAdV-species, the great apes have AdVs that are more related to their human-derived counterparts [16,17]. Moreover, some of the human species may, in fact, be of ape origin [18]. While the monkey-derived AdVs are most often included in one of nine simian AdV (SAdV) species SAdV-A through SAdV-I, the ape-derived AdVs are sometimes included in the most related HAdV-species HAdV-A, -B, -C, -E, or -G (National Center for Biotechnology Information (NIH)/GenBank). Note however, that there is no organization that formally attributes AdV isolated from non-human hosts to a specific species. The Human Adenovirus Working Group only classifies new types isolated from humans, and the International Committee on Taxonomy of Viruses (ICTV; https://talk.ictvonline.org accessed on 21 August 2022) decides on the taxonomy of viruses to the species level and is not currently addressing the classification below this level. 

Non-human primate AdVs have been explored as gene-transfer vectors to which low levels of neutralizing immunity exists in the human population [16,19]. To expand the collection of available non-human primate AdVs, we isolated new AdVs from healthy western lowland gorillas, bonobos, chimpanzees, and orangutans kept in Dutch zoos [20]. This yielded 41 unique isolates, as based on sequencing of the hypervariable regions 1–7 of the hexon gene. Of the 41 unique isolates, 29 are most similar to the HAdV-C species. Here we provide the results of the whole genome analyses of 10 of the 29 isolates that phylogenetically cluster with the HAdV-C species. We compare these sequences with previously isolated non-human primate AdVs with high similarity to HAdV-C. We provide evidence that while these new isolates cluster with the genomes of the human-derived HAdV-C, their genomes differ considerably in lengths, nucleotide composition, the amino-acid sequences of the encoded capsid proteins, the replication proteins, and the AdV proteases. The phylogenetic analyses also reveal a strict clustering of the viruses depending on the host species from which they have been isolated (viz. bonobo, chimpanzee, and gorilla). Taken together, these data suggest that the AdVs from gorilla and from the combined bonobo and chimpanzee clades may better be included in a new SAdV species and not in HAdV-C.

## 2. Results

### 2.1. Genome Analyses of New Ape Adenoviruses 

The isolation of the new AdV isolates analyzed here was described previously [20]. Out of 41 new and unique isolates, 29 clustered with the HAdV-C species in a comparison of the nucleotide sequences of region coding for their hypervariable regions 1–7 of the hexon gene. Here we present analyses of the full genome sequences of ten of these isolates. 

**Figure 1:**
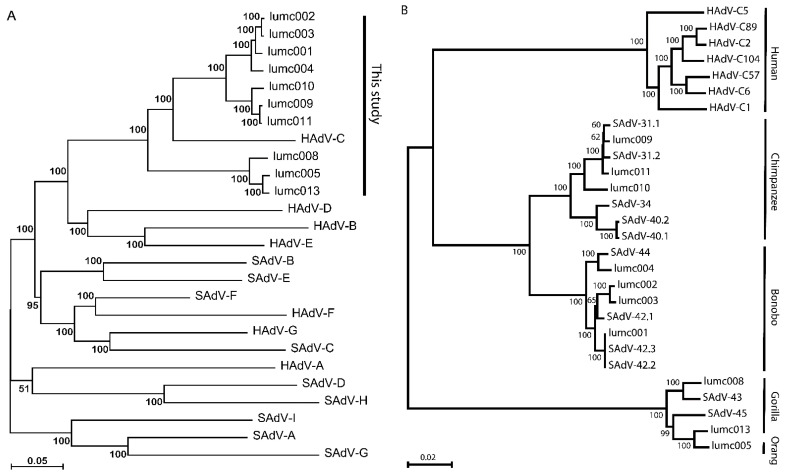
(**A**) Phylogeny reconstruction based on the full genome sequences of the new AdVs and representatives of all current human and simian *Mastadenovirus species*. Each of the species is indicated by the formal species name. The new AdV isolates lumc001–005, and lumc008–011, and lumc013 and their NCBI-GenBank accession numbers are summarized in Table 2. The representatives of each of the species, and their NCBI-GenBank accession numbers are indicated in Table 1. (**B**) Phylogeny reconstruction based on the full genome sequences of the new AdVs, the most similar simian AdVs, and the human-derived HAdV-C types. The host species from which the AdVs were isolated are indicated on the right-hand side.

To confirm the similarity of the new Ad-lumc viruses with the HAdV-C, we retrieved from the NCBI GenBank the genome sequences of representative members of each of the simian (SAdV-A through SAdV-I) and human (HAdV-A through HAdV-G; see Table 1) Mastadenovirus species. These full genome sequences were used to generate a phylogenetic tree based on the evolutionary relationship (Figure 1A). This tree confirms that the new Ad-lumc viruses are all most closely related to HAdV-C and thereby support our initial hexon-based species assignment. Next, we retrieved the full genome sequences of other primate-derived AdVs that have high sequence similarity with HAdV-C. These viruses and their accession numbers are summarized in Table 2. 

In all further analyses we included the full sequences of examples of all known HAdV-C types isolated from humans (viz. HAdV-C1, C2, C5, C6, C57, C89, and C104), and the primate-derived AdVs that cluster with HAdV-C (viz. SAdV-31.1, 31.2, 34, 40.1, 40.2, 42.1, 42.2, 42.3, 43, 44, and 45). The evolutionary relationship is represented as a phylogenetic tree of all AdVs clustering to HAdV-C in Figure 1B.

There are consistent differences in the nucleotide composition between the AdVs isolated from the different primate hosts (Table 2). The genomes of HAdV-C AdVs isolated from humans have a G + C percentage of 55.3 ± 0.1%, while the AdVs from apes have more GC-rich genomes (bonobos 60.5 ± 0.1%, chimpanzees 60.5 ± 0.2%, gorillas 57.8 ± 0.2%, and the virus from orangutans 56.8%). All these values fall outside the G + C percentage range observed in human-derived HAdV-C genomes. 

In addition, these non-human primate AdVs have significantly larger genome sizes than the human-derived HAdV-C. Especially the average genome sizes of the AdVs isolated from chimpanzees (37,792 ± 67 bp) and bonobos (37,754 ± 62 bp) considerably exceed the average size of the human-derived HAdV-C genomes (35,901 ± 83 bp). The gorilla (37,157 ± 51 bp) and orangutan (37,034 bp)-derived AdV genomes are smaller than those of bonobo- and chimpanzee-derived AdVs, but still exceed the genome sizes of the human-derived HAdV-C viruses by at least 1000 base pairs.

In the coding regions for the major capsid proteins, the increased lengths of the RGD loops of the penton-base proteins are remarkable in the non-human primate-derived AdVs (Table 2). To compare these loops, we aligned the penton-base amino-acid sequences of the AdVs and marked the sequences of the RGD loop, as defined by Madisch et al. [21]. These authors compared the lengths of the RGD loops of the HAdV types 1–51 and noted that the lengths of the RGD-loops in the human-derived HAdV-C types C1, C2, C5, and C6 (101 ± 2 amino acids on average) exceed those of the RGD loops in all non-HAdV-C types. In the non-human primate-derived viruses clustering to HAdV-C, the loops are longer in viruses isolated from gorillas (188 ± 16 amino acids) and orangutans (163 amino acids) than in viruses from chimpanzees (123 ± 4 amino acids) and bonobos (117 ± 2 amino acids). Nevertheless, also in the AdVs from the latter hosts, the loop lengths exceed the RGD-loop lengths of the human-derived HAdV-C viruses. The lengths of the proline-rich region, and of the hypervariable region 1 of the penton-base proteins (as defined in [21]) did vary as well but to a much lesser extent than those of the RGD loop (Table 2). 

### 2.2. Molecular Phylogeny of AdV Proteins

**Figure 2:**
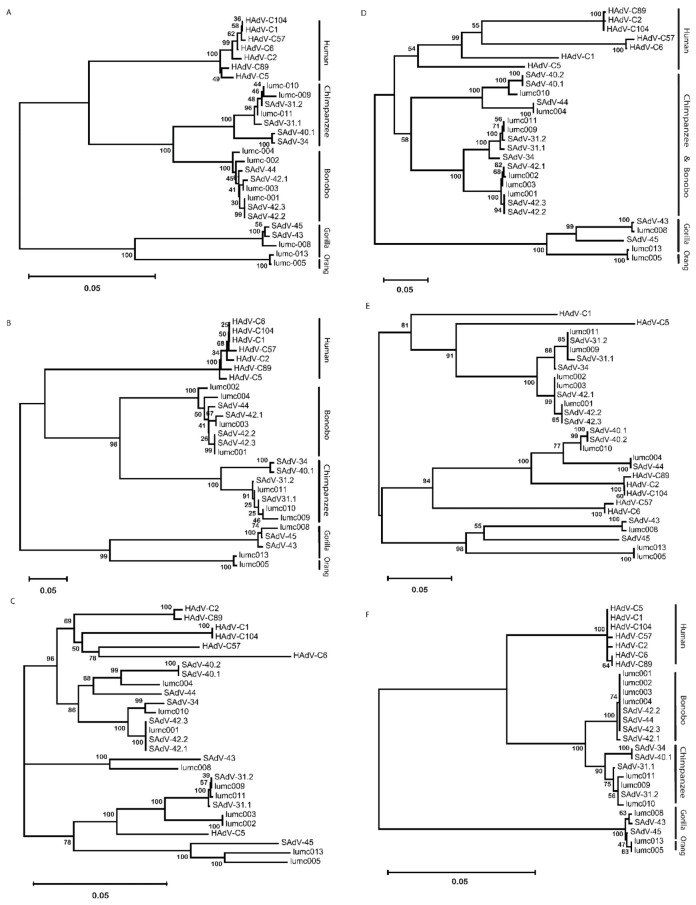

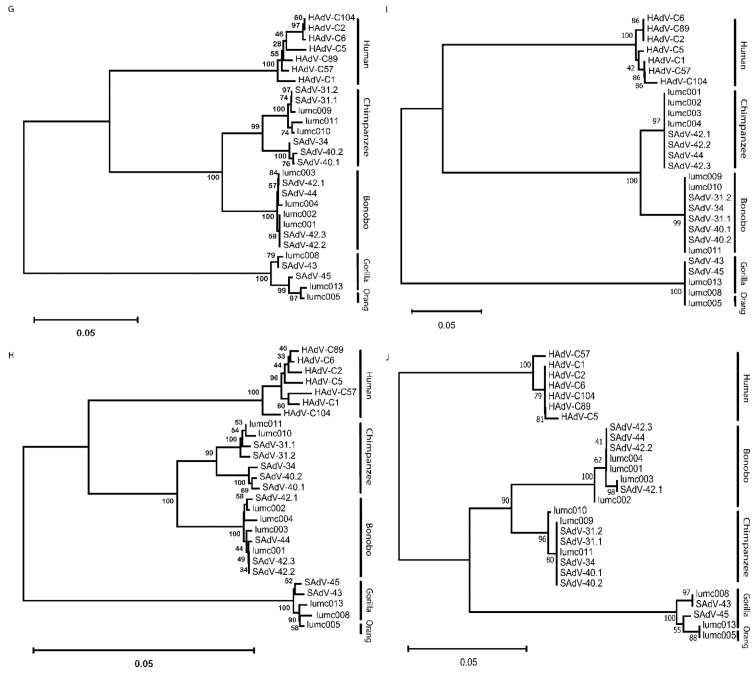
Phylogeny reconstruction based on the amino-acid sequences from (**A**) the penton-base protein, (**B**) the penton-base ‘RGD’ loop, (**C**) the hexon protein, (**D**) the fiber, (**E**) the fiber knob, (**F**) protein IIIa, (**G**) the single-stranded DNA-binding protein, (**H**) the AdV polymerase, (**I**) the minor capsid protein IX, and (**J**) the AdV protease protein. The host species from which the AdVs were isolated are indicated on the right-hand side in the cases where the phylogenetic clades coincide with the host species from which the viruses were isolated.

To study the similarity of the amino-acid sequences of the proteins from the AdVs of the different hosts, we used multiple amino-acid sequences and phylogeny inferences. This demonstrates that the penton-base sequences cluster in a host-specific manner (Figure 2A). Comparison of the sequences of the RGD-loop only yielded a very similar pattern (Figure 2B). Remarkably, the Ad-lumc005 virus that was isolated from an orangutan clusters with the sequences from Ad-lumc013 isolated from a gorilla and, albeit more distantly, also with the penton-base sequences of SAdV-45, SAdV-43, and Ad-lumc008 which are also isolated from gorillas. Similar phylogenetic analyses were performed with the amino-acid sequences of the constant regions of the hexon proteins (Figure 2C), and the fibers (Figure 2D), and fiber-knob sequences (Figure 2E). These analyses yielded very similar patterns, and again, the different clusters match the host species from which the viruses were isolated, although in these trees the sequences from the chimpanzee and bonobo AdVs are not strongly separated. Analyses of the amino-acid sequences of the minor capsid proteins IIIa (Figure 2F) showed a similar picture, in which the sequences from viruses of humans, bonobos, chimpanzees, and gorillas are clustered and the clades are well separated. This pattern was further confirmed for the AdV single-stranded DNA-binding protein DBP (Figure 2G), the AdV polymerase (Figure 2H), the minor capsid protein IX (Figure 2I), and the AdV protease (Figure 2J). In each of the analyses the proteins from the Ad-lumc005 virus isolated from orangutans clustered with the viruses from gorillas. Taken together, the phylogenetic reconstructions show a clear tendency to group these AdVs into separate clades in a host-specific manner.

### 2.3. Recombination

Homologous recombination between AdVs is relatively frequent and contributes to the genetic diversity in the human-derived HAdV-C types. To study whether recombination is also evident between AdVs from the non-human primates, we used similarity plots and bootscan analyses for the AdVs from each of the host species. We found evidence of homologous recombination within each of the chimpanzee, bonobo, and gorilla AdV clades. This is in accordance with previous reports showing that, similar to the human-derived AdV genomes, in non-human primate AdVs homologous recombination is a major contributor to their genetic diversity [17,18,22,23].

### 2.4. The Origin of the AdV Isolate from Orangutan

The isolation of an AdV from a stool sample of an orangutan was surprising, as so far, no AdV was isolated from this species, although serology [24] and PCR [25] had provided evidence for the occurrence of AdVs in this species. The AdV isolated from orangutan, Ad-lumc005, has a genome length, nucleotide composition, and RGD-loop length similar to the AdVs isolated from gorillas. Sequence alignment and nucleotide similarity plots demonstrated that the Ad-lumc005 virus is more similar to the gorilla AdVs at the genome level than to the AdVs from chimpanzees, bonobos, and humans (Figure S1). In the phylogenetic analyses of viral proteins, the Ad-005 virus also consistently clusters with the protein sequences of gorilla-derived AdVs (Figure 2).

**Figure 3:**
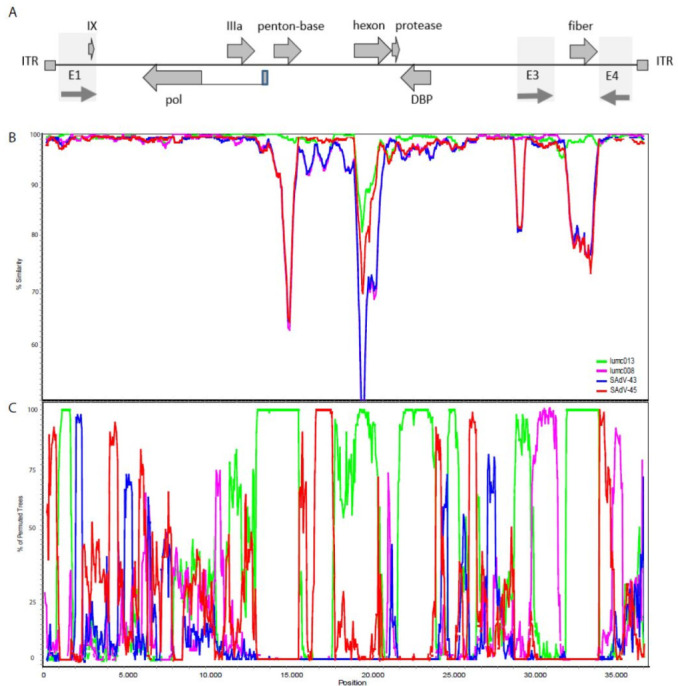
(**A**) Schematic representation of the AdV genome indicating the inverted terminal repeats (ITR), a number of the protein-coding open-reading frames, and the early regions E1, E3, and E4. (**B**) Nucleotide similarity plots constructed with Simplot representing the number of nucleotide differences per site between the AdV-lumc005 isolate and each of the gorilla-derived isolates that clusters with HAdV-C. The % similarity is calculated on the y-axis and the x-axis illustrates the nucleotide position on the genome. The AdV lumc013 is indicated in green, lumc008 in purple, the SAdV-43 virus in blue, and SAdV-45 in red. (**C**) Bootscan analysis of the orangutan-derived AdV-lumc005 genome with the gorilla-derived AdVs lumc013 (green), lumc008 (purple), SAdV43 (blue), and SAdV45 (red). The window size was 500 bp, the step size was 50 bp, and the bootstrap replication number was 100. The distance model was Kimura (2-parameter), and the tree model was the neighbor-joining algorithm. Regions of the curves where more than 70% of the permutated trees are derived from a single virus are indicative of recombination. This suggests that the orangutan-derived lumc005 AdV acquired the genes encoding penton-base, the hexon, the single-stranded DNA-binding protein, and the fiber from the gorilla-derived Ad-lumc013.

This led to the hypothesis that these viruses may be genetically related. Therefore, we analyzed its phylogenetic relationship to the gorilla AdVs. Similarity plots were used to compare the genome of the Ad-lumc005 virus with the sequence of the human-, gorilla-, bonobo-, and chimpanzee-derived genomes. This demonstrated that the genome of this virus is most similar to the gorilla-derived viruses. Similarity plots and bootscan analyses were used to study the relationship of Ad-lumc005 with the four gorilla-derived AdVs (Figure 3). Both analyses suggest that Ad-lumc005 is a mosaic recombinant virus related to Ad-lumc008, Ad-lumc013, SAdV43, and, to a lesser extent, SAdV45. This suggests that the Ad-lumc005 virus may not originate from the orangutan, but rather is a gorilla AdV.

## 3. Discussion 

To expand the collection of available AdVs from non-human primates, we isolated a series of new AdVs from great apes and analyzed their genome sequences. In the simian-derived AdVs with similarity with the human-derived HAdV-C viruses there are consistent differences in their nucleotide composition, genome size, and in the protein structure, e.g., the lengths of their RGD-loops in penton-base. This RGD loop physically extends from the penton and, in most AdV types, contains an arginine–glycine–aspartic acid (RGD) motif. This motif can interact with αvβ3 or αvβ5 integrins and this interaction facilitates endocytosis of the AdV particle [26,27,28]. The difference in the primary sequence and the spatial structure of the RGD loop may alter the topology and accessibility of the RGD peptide and thereby affect the binding to integrins [29]. It is tempting to speculate that this could underly the distinct gastrointestinal tropism of the ape-derived AdVs compared to their human-derived counterparts [25]. Nevertheless, for human-derived AdVs, the length of the RGD loop alone does not seem to be associated with enteric tropism [21]. Therefore, it would be interesting to identify the length of the RGD loop in other species of ape-derived AdVs, and to study its molecular structure and binding mechanism.

One of the surprising findings was the isolation of an AdV from fecal samples of the orangutan. So far, we are not aware of the isolation of replicating AdVs from this host, although PCR analyses of fecal samples of orangutans held in captivity yielded AdV sequences in 17 out of 27 samples tested [25]. In all our analyses, sequences from the Ad-lumc005 isolated from the orangutan clustered with the gorilla-derived AdVs. The high similarity, as well as the bootscan evidence of multiple recombinations, suggest contact between these viruses. This is unlikely to have occurred in nature in light of the non-overlapping geographical ranges in which gorillas and orangutans occur. It is therefore more likely that a gorilla-derived AdV was transmitted to the orangutan in captivity. This may be a feasible route, as AdV immunity has been reported to prevail in 18/44 (40%) of semi-captive orangutans and in only 3/54 (5%) of free-ranging animals [24]. This difference in frequency could suggest that AdV transmission can occur at increased population densities, probably by aerosol or fecal-oral transmission. We cannot formally exclude a scenario in which the isolation of this virus is the result of laboratory contamination. However, we consider this unlikely, as we isolated this virus twice from orangutan stool samples, and none of the other gorilla AdVs that we isolated and handled had a sequence that is identical to the Ad-lumc005 isolate. Nevertheless, we hesitate to label Ad-lumc005 as a genuine orangutan AdV and rather consider it as an AdV with a gorilla origin.

The phylogeny inference leads to distinct grouping of the AdVs. The human-derived HAdV-C viruses and those from gorilla and bonobo/chimpanzee all form distinct and separate clades. The branch lengths for hallmark proteins, such as AdV DNA polymerase, protease, DBP, protein IIIa, protein IX, fiber, and penton-base all exceed a value of 0.05 between the clusters of human, gorilla, and chimpanzee/bonobo viruses. 

The G + C percentages of the human-derived and the gorilla-derived AdVs differ by 2.5% and the human and combined group of bonobo- and chimpanzee-derived AdVs by 5.3%. This is at or above the species-demarcating differences of maximally 2.5% that were found within different simian AdV species [30]. This indicates that the human AdVs, the gorilla AdVs, and the group combining the chimpanzee and bonobo AdVs form distinct evolutionary clades. Note that the G + C percentages of the HAdVs reported here are slightly lower than the values of 57 to 59% often reported for HAdV-C in the literature. This difference is probably caused by the different methods used to establish the G + C percentages. Whereas the classical values are based on measurements of the buoyant density and melting temperatures of isolated AdV DNA [31], in this study we relied on nucleotide counts of their genome sequences. 

These analyses, combined with their distinct genome sizes, and the differences in nucleotide composition warrant the human AdVs, the gorilla AdVs, and the combined group of bonobo and chimpanzee AdVs to be considered as clades of different taxa. These data confirm and extend the results of Kang and collaborators who used full genome sequence analyses of a large set of human and simian adenoviruses to provide a proposal for a more consistent and revised taxonomy for the primate adenoviruses [32]. Based on the observation that many of the clades encompass AdV isolates from humans as well as from non-human primates, they propose the designation of all AdVs isolated from humans and from non-human primates as ‘primate adenoviruses’ (PrAdV). This clade includes all AdVs isolated from humans and from simians. The human-, gorilla-, chimpanzee-, and bonobo-derived AdVs that are conventionally grouped in the HAdV-C species are proposed to be reclassified as three distinct species: PrAdV-C (the human-derived HAdV-C types such as C1, C2, C5, C6, and C57), PrAdV-K (gorilla-derived isolates as SAdV-43 and SAdV45), and PrAdV-S (bonobo- and chimpanzee-derived isolates such as SAdV-34 and SAdV-44) [32]. This PrAdV classification is fully supported by the analyses of the HAdV-C isolates presented in our study. In this classification, our orangutan-derived adenovirus Ad-lumc005 would be included in the PrAdV-K species. Whether this revised classification will be formally adopted is for the ICTV to decide.

## 4. Materials and Methods 

### 4.1. Cell Lines 

The HAdV-C5 E1 transformed human embryonic retinoblast cell line HER911 was used for the isolation and propagation of the non-human primate-derived AdVs as described [20,33]. The HER911 cells were cultured in high glucose Dulbecco’s Modified Eagle’s Medium (DMEM, Gibco, Carlsbad, MA, USA) supplemented with 8% fetal bovine serum (FBS, Invitrogen, Carlsbad, MA, USA) and 1% Penicillin-Streptomycin (P/S, Gibco). All cells were cultured in an atmosphere of 5% CO_2_ at 37 °C. 

### 4.2. Virus Isolation 

Fecal samples were obtained from Gorilla gorilla gorilla (Western lowland gorilla), Pan troglodytes (chimpanzee), Pan paniscus (bonobo), and Pongo pygmaeus (Bornean orangutan) held in captivity in Dutch zoos. The viruses were isolated as described [23] with the modifications described previously [20]. Briefly, aliquots of 250–500 mg feces were dispersed in 5 mL phosphate-buffered saline without Ca^2+^ and Mg^2+^ (PBS^−^) and the suspension was cleared by centrifugation. The cleared suspension was passed twice through 0.45 µm Acrodisc^®^ syringe filters (PN4148, Pall Life Sciences, New York, NY, USA). From each filtered sample, 100 µL and 10 µL aliquots were added to near-confluent cultures of HER911 cells grown in 6-well plates (Thermo Fisher Scientific, Leiden, The Netherlands) in DMEM with 8% FBS supplemented with a cocktail of antibiotics to suppress microbial growth. The cultures were inspected every other day for signs of cytopathic effects (CPE) for up to 4 weeks post infection. Upon clear signs of CPE, cells were harvested, and the viruses were released from the cells by three cycles of freeze-thawing (F/T), followed by pelleting the cellular debris by centrifugation. An aliquot of the supernatant was added to a fresh near-confluent culture of HER911 cells in DMEM with 8% FBS and the antibiotics cocktail. When CPE was nearly complete, the medium with the cells was collected and the cells were lysed by three cycles of F/T. The lysates were cleared by centrifugation and stored for further use at −20 °C.

### 4.3. Hexon Sequencing

For isolation of hexon hypervariable regions of DNA for sequencing, 100 µL of virus-containing supernatant was added to near-confluent cultures of HER911 cells. Once CPE became apparent, cells and medium were collected and used for a modified Hirt DNA extraction procedure optimized for AdV DNA isolation as described [20]. Approximately 10 ng of DNA was used for PCR amplification of the hexon hypervariable region (HHVR) 1–7 using the following primers: 5′-CAGGATGCTTCGGAGTACCTGAG-3′ (forward primer), and 5′-TTGGCNGGDATDGGGTAVAGCATGTT-3′ (reverse primer). In these sequences ‘N’ is used to represent any base, ‘D’ indicates A, G, or T, while ‘V’ denotes A, C, or G. Standard PCR reactions were performed (30 s 55 °C, 1 min 72 °C, 1 min 95 °C, 30 cycles) and the sequences of the amplicons were determined by Sanger sequencing at the Leiden Genome Technology Center.

### 4.4. Next Generation Sequencing

From ten of the new ape AdVs, DNA was isolated by a modified Hirt extraction for sequencing on an Illumina platform by BaseClear B.V. (Leiden, the Netherlands). The viral genomes were assembled de novo and viral genes were annotated by comparing the DNA sequences with the AdV genome sequences annotated in the NCBI GenBank nucleotide databases (https://www.ncbi.nlm.nih.gov/nuccore/ accessed 10 September 2021). All Ad-lumc full genome sequences are available in GenBank (Table 2).

### 4.5. Genome Analyses

The collection of AdV genome sequences was imported from NCBI GenBank into a Vector NTI Advance 11.5.0 (Thermo Fisher Scientific, Leiden, Netherlands) database. From this database, AdV sequences were exported as a FASTA format file and read into MAFFT 7.427 for Windows and aligned with the FFT-NS-2 strategy [34]. The output was read into MEGA-X 10.2.4 for phylogeny inference by neighbor-joining tree generation [35]. Simplot version 3.5.1 [36] was used for similarity plots with the following parameters: Window 1000 bp, Step 100, GapStrip: On, Kimura (2-parameter), and T/t: 2.0. For recombination analyses by bootscanning, we used the settings: Window 500 bp, Step 50, GapStrip: On; Reps 100, Kimura (2-parameter), and T/t: 2.0, for the Neighbor-Joining algorithm. From relevant sequences, the percentage of G + C nucleotides was calculated using the GC Content Calculator (en.vectorbuilder.com accessed 9 January 2022). For editing aligned sequences, the Base-by-Base multiple alignment editor was used [37].

## Figures and Tables

**Table 1:** The Mastadenovirus species, the representative types, and NCBI GenBank accession numbers.

AdV Species	Type	Accession Number
SAdV-A	SAdV-3	NC_006144
SAdV-B	SAdV-08	NC_028113
SAdV-C	BaAdV-3	KC693023
SAdV-D	SAdV-13	NC_028103
SAdV-E	SAdV-16	NC_028105
SAdV-F	SAdV-18	FJ025931
SAdV-G	SAdV-20	NC_020485
SAdV-H	SAdV-DM2014	NC_025678
SAdV-I	SAdV-WIV19	KX505867
HAdV-A	HAdV-A12	KX868289
HAdV-B	HAdV-B3	NC011203
HAdV-C	HAdV-C2	AC_000007
HAdV-D	HAdV-D8	KP016723
HAdV-E	HAdV-E4	AY458656
HAdV-F	HAdV-F40	KU162869
HAdV-G	HAdV-G52	DQ923122

**Table 2:** The *Mastadenovirus* types, their hallmarks, origins, and the accession numbers of their genome sequences.

Virus Isolate	Genome Length	Percentage G + C Basepairs	Hexon Length	Penton-Base Length	Penton-Base RGD-Loop	Penton-Base HVR1 Loop	Penton-Base Proline-Rich Region	Host Species	GenBank Accession Number
SAdV-42.2	37,820	60.4	957	586	120	11	27	Bonobo	FJ025902
Ad-lumc001	37,820	60.5	957	587	120	11	27	Bonobo	MZ882379
SAdV-42.3	37,820	60.6	957	586	120	11	27	Bonobo	FJ025925
Ad-lumc002	37,702	60.4	955	584	118	11	27	Bonobo	MZ882389
Ad-lumc003	37,724	60.4	955	582	116	11	27	Bonobo	MZ882380
SAdV-42.1	37,786	60.3	957	581	115	11	27	Bonobo	FJ025903
Ad-lumc004	37,647	60.6	963	581	115	11	27	Bonobo	MZ882381
SAdV-44	37,711	60.6	962	581	115	11	27	Bonobo	FJ025899
Ad-lumc009	37,885	60.6	954	592	128	11	25	Chimpanzee	MZ882384
SAdV-40.2	37,729	60.3	960	596	126	11	31	Chimpanzee	FJ025926
SAdV-31.1	37,828	60.6	954	589	125	11	25	Chimpanzee	FJ025906
SAdV-31.2	37,860	60.6	954	589	124	11	25	Chimpanzee	FJ025904
SAdV-34	37,799	60.2	958	593	123	11	31	Chimpanzee	FJ025905
SAdV-40.1	37,718	60.3	960	593	123	11	31	Chimpanzee	FJ025907
Ad-lumc010	37,738	60.7	958	581	117	11	25	Chimpanzee	MZ882385
Ad-lumc011	37,780	60.6	954	579	115	11	25	Chimpanzee	MZ882386
Ad-lumc008	37,200	58.0	959	656	199	8	23	Gorilla	MZ882383
SAdV-45	37,152	57.6	953	651	194	11	27	Gorilla	FJ025901
SAdV-43	37,188	58.0	955	651	194	6	23	Gorilla	FJ025900
Ad-lumc013	37,087	57.6	947	622	164	7	23	Gorilla	OM807070
HAdV-C57	35,818	55.2	959	574	102	11	32	Human	HQ003817
HAdV-C1	36,001	55.3	964	574	102	11	32	Human	AC_000017
HAdV-C104	35,933	55.3	964	574	102	11	32	Human	MH558113
HAdV-C6	35,758	55.4	978	574	102	11	32	Human	FJ349096
HAdV-C2	35,937	55.2	968	571	99	11	32	Human	AC_000007
HAdV-C5	35,938	55.2	952	571	99	11	32	Human	AC_000008
HAdV-C89	35,923	55.2	969	570	98	11	32	Human	MH121114
Ad-lumc005	37,034	56.8	947	621	163	7	23	Orang utan	MZ882384

## Data Availability

Any data relating to this study and are that not already available in Genbank will be made available upon reasonable requests.

## Supplementary Materials

The following supporting information can be downloaded at: https://www.mdpi.com/article/10.3390/ijms23179832/s1.
