# Supplementary material for: Genome Analyses of Ten New Ape Adenoviruses with Similarity to Human Mastadenovirus C
Source: Int J Mol Sci. 2022 Aug 30;23(17):9832. doi: 10.3390/ijms23179832 (PMC9456536; doi:10.3390/ijms23179832)
Supplement: Supplementary file 1 [file ijms-23-09832-s001.zip › ijms-1894120-supplementary.pdf]

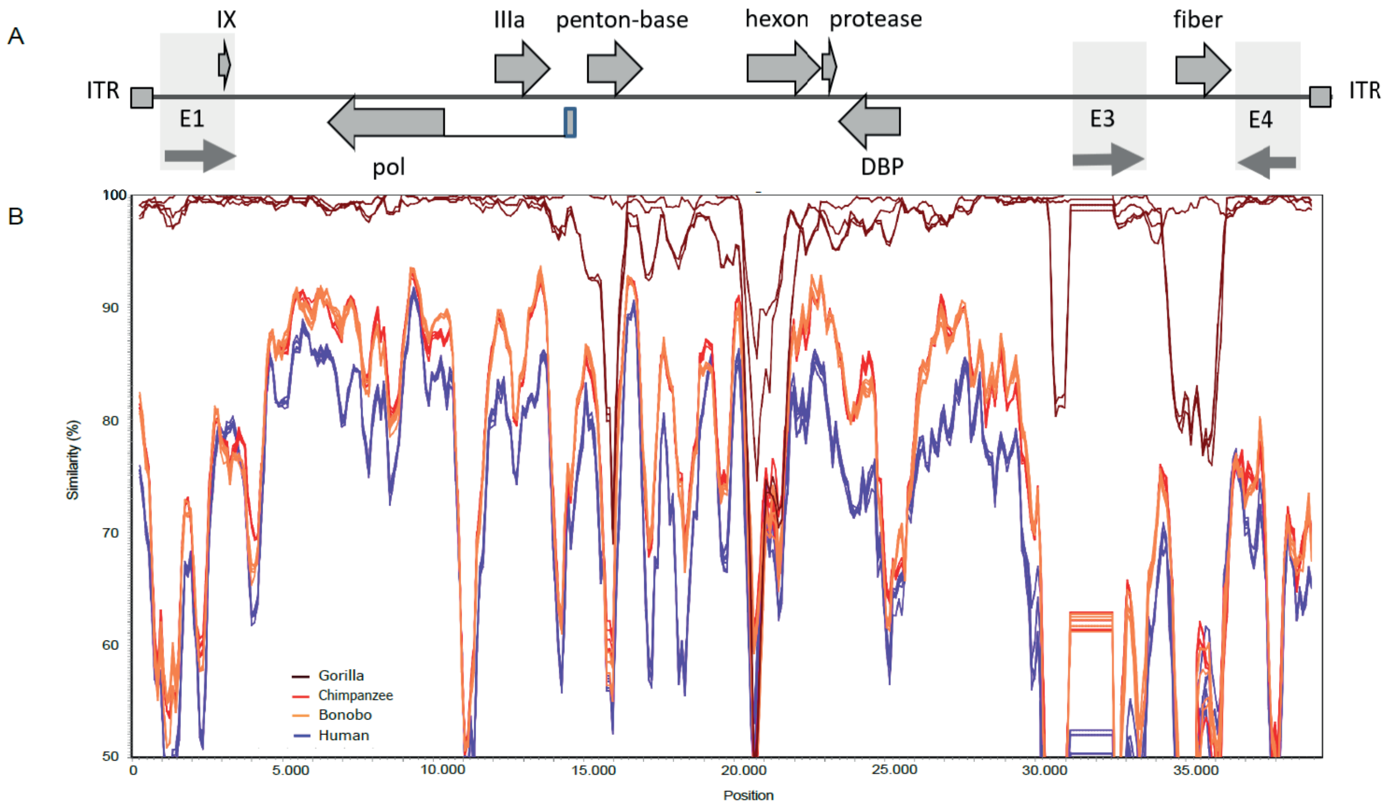

Figure S1
(A) Schematic representation of the AdV genome indicating the inverted terminal repeats (ITR), a number of the protein-coding open-reading frames, and the early regions E1, E3 and E4.
(B) Nucleotide similarity plots constructed with Simplot representing the number of nucleotide differences per site between the AdV-lumc005 isolate and each the human (blue), bonobo (orange), chimpanzee (red), and gorilla-derived (brown) isolates that clusters with HAdV-C. The % similarity is calculated on the y-axis and the x-axis illustrates the nucleotide position on the genome.
